# The Use of the Blockchain Technology and Digital Watermarking to Provide Data Authenticity on a Mining Enterprise

**DOI:** 10.3390/s20123443

**Published:** 2020-06-18

**Authors:** Oleg Evsutin, Yaroslav Meshcheryakov

**Affiliations:** 1 Department of Cyber-Physical Systems Information Security, HSE Tikhonov Moscow Institute of Electronics and Mathematics, National Research University Higher School of Economics, 123458 Moscow, Russia; 2 Laboratory of Cyber-Physical Systems, V. A. Trapeznikov Institute of Control Sciences of Russian Academy of Sciences, 117997 Moscow, Russia; 3 Department of Complex Information Security of Computer Systems, Tomsk State University of Control Systems and Radioelectronics, 634050 Tomsk, Russia; meshcheryakovye@gmail.com

**Keywords:** mining industry, sensor controls, cyber-security, blockchain, digital watermarking

## Abstract

Prompt development of information technology has made an essential impact on many industries. There appeared a concept “Industry 4.0” symbolizing the fourth industrial revolution. The given concept is closely connected with such promising technologies as the Internet of Things, blockchain, fog computing, Big Data. In the present research, the sphere of the mining industry is examined. We discuss the possibility to increase the efficiency of mining enterprises at the expense of the development of common information space based on modern digital technologies. We analyze security problems at the level of data flow between the participants of the production process on a mining enterprise. We define the problem of providing the reliability of data on the production course on mining enterprise in the conditions of the possible connection loss between the control center and separate technological units. We offer a new approach to the solution of the given problems, based on the technology of blockchain and digital watermarking. The computing experiment is conducted presenting a possibility to implement the offered approaches on common models of microcontrollers.

## 1. Introduction

The prevailing direction of the development of the world mining industry in the immediate future is considered to be open-cut mining of deposits as it allows one to ensure the best economic indicators. The share of open-cut mining in ore mines, opencast mines and open-casts is over 80% of world mining production. In Russia, 91% of iron ores, more than 70% of nonferrous metal ores, more than 60% of coal is extracted by open-cut mining. At the same time, all over the world the depth and scales of open-cut mining are increasing [1,2,3,4].

The increase of open-cut mining efficiency can be reached in two ways:

- at the expense of application of new types of mining service machines and transport equipment;
- at the expense of the implementation of common information space with modern digital technology at mining enterprises.

The first way allows increasing the efficiency of open-cut mining at the level of the primal production process. The second way allows regulating the activity of divisions and employees, increasing the speed of decision-making, automating the data flow in the enterprise, and considerably increasing the efficiency of automation.

The key element of common information space of a mining enterprise is the automated decision support systems. They process Big Data flow coming from various sources. The timely information on the current condition of the enterprise is necessary for the correct operation of such systems at all levels.

The development of common information space of a mining enterprise is a major problem including a number of aspects and subtasks. Our research is concentrated on such aspect of the general problem as protection of data on the production course. Primarily, this refers to the data on the actions of technological units.

In the case of the industry under consideration, the problem of data protection has a number of distinctive features which define the urgency of our paper. The main feature consists in the fact that on an opencast mine, there is not always a connection with the service units because of the features of the landscape. Therefore, the control unit can receive the distorted information on the actions of technological units, or during some time not to receive it at all. On the one hand, it negatively affects the quality of management. Because of the connection loss, the automated systems not always can correctly direct the service support transport to a certain excavator or clearly define if a support service machine belongs to a certain group. On the other hand, it allows unfair workers to hide the actions violating industrial regulations. For example, in the case of big enterprises, where is a set of working sites, the problem of friend-or-foe identification is urgent. Technological transport machines can come to the wrong working sites for loading.

Thus, the main objective of this paper is to solve the problem of providing the reliability of data on the production course on a mining enterprise in the conditions of possible connection loss between the control center and separate technological units. As a whole the given research brings the following contribution:

- the problems of security at the level of data flow between the participants of the production process on a mine are analyzed and the key problems requiring the immediate solution are selected.
- the approach to transaction reliability provision in the common information space of a mine, based on the blockchain technology is offered. A transaction is understood as any significant event, occurred to technological units in the course of their work. The format of transactions is defined and the list of transactions for two basic types of technological units is generated.
- the approach of digital watermarks to providing the authentication of the sensor data generated by service support transport is offered. This approach is supplemented blockchain and ensures the additional data binding to the conditions of their generating.

The rest of the given article is structured as follows. The review of previous research on the topic is presented in Section 2. Section 3 is devoted to the ways of the solution of the problems arising at the development of common information space at a mining enterprise. The results of some computing experiments and their discussion are presented in Section 4. The conclusion sums up the research.

## 2. Review of Related Works

One of the key problems of establishing common information space on a mining enterprise is data protection. A distinctive feature of the given problem is the necessity to record the actions performed by support service vehicles in the conditions of the lack of reliable communication channels between the machinery units and the control unit. The perspective technology for the solution of the given problem is the blockchain technology.

The blockchain is a decentralized technology that ensures the integrity of transactions without the participation of a third party. Blocks (smart contracts) describe the executed transactions and communicate with each other through cryptographic transformation. Intentional alteration of the block will compromise all other existing blocks in the chain, and then all blocks are to be processed again to become valid. It makes the process of hacking the blockchain quite difficult [5].

Currently, blockchain technology is commonly applied in various areas of the industry. The main value of the given technology consists in the fact that it allows one to ensure the confirmation of various transactions executed in an untrusted environment. Numerous studies prove the importance of blockchain technology for the fourth industrial revolution (Industry 4.0), for example, [6,7,8].

Besides, within the limits of Industry 4.0, blockchain is developing along with other perspective technologies of our time. They include the Internet of Things [9,10], Big Data [11], augmented reality [12,13,14], fog computing [10,14,15], and robotic science [16]. As a whole, blockchain is examined as one of the key technologies of the industrial Internet of Things, promoting the modernization of traditional factories into modern intelligent factories using the last achievements in the field of digital technology. 

Let’s note some examples of the modern studies offering definite scientific and engineering solutions connected to blockchain application.

The essential part of known papers concerns the problem of safe management of various actives. It is quite natural; after all, the first application of blockchain was connected with bitcoin cryptocurrency. The further development of blockchain also went in this direction; the market of cryptocurrency appeared which now plays an appreciable role in the life of society.

However, eventually, the number of application of the blockchain technology has considerably increased. It is possible to select three types of studies devoted to the blockchain application.

The studies of the first type are devoted to the management of delivery chains with blockchain technology. Such research is of a more general character, it does not select any certain area but offer a common solution on the safe management of delivery with the use of blockchain and discusses some aspects of the given problem.

So, paper [17] presents a classification of limitations impeding the implementation of blockchain technology in the management of delivery chains. Some problems of overcoming such limitations are presented in [18]. In both cases, the definite active is not specified. The studies that examine a very wide list of services and goods refer to the given type. Article [19] describes real-life cases of blockchain application for tracing of raw material sources, components, or spare parts in various industries. 

The studies of the second type solve the problem of safe management of any certain version of actives or a type of service. Currently, the number of such applications has become rather high. The blockchain is applied to control the sales of municipal energy [20], fuel [21], computing resources [22], and software [23].

All listed studies are united by the fact that there is a commodity-money exchange. Therefore, offered blockchain solutions in many respects inherit the ideas of cryptocurrency.

At last, the third type of studies is devoted to the problem of entrusted interaction between a set of devices. An indicative example is the area of the connected vehicles [24,25,26]. Such papers are not numerous; however, this area has started to develop actively.

Another promising direction of research in the field of data protection is based on the embedding of hidden multipurpose information sequences into transmitted and stored digital data. In the given direction, there are methods of digital steganography and methods of digital watermarking. Methods of digital steganography are designed for establishing hidden channels of confidential information transmission in digital objects. Methods of digital watermarking are used for authentication of digital objects and their owners.

Usually, multimedia data are used as containers for additional information embedding. However, for the industrial sector, the work with data of a different kind is urgent. In particular, it is a question of data that is gathered from various sensors and is transmitted by communication channels.

Currently, rather quickly developing is the research area connected to the application of digital steganography methods and digital watermarking to non-media data. There have appeared a number of papers devoted to the hiding of information in the data of wireless sensor networks, which is the most urgent for the mining sector. As an example, we will note the papers [27,28,29,30,31,32,33,34]. Within the limits of the present research, similar methods can be used for the authentication of mining service machine data.

Blockchain and watermarking are directed on the solution of different problems of cyber-security. Their joint use will potentially allow achieving a higher security level than when using the given technologies separately. This idea has already found the reflection in the previous studies; however, mainly only in one direction connected to the problem of digital rights management [35,36,37]. Therefore, joint use of the given technologies in other applications is a perspective direction of the research, the development of which would allow bringing the contribution to the important area of cyber-security.

Thus, the executed review allows us to draw the following conclusions. 

Modernization of traditional mining facilities towards the intelligent manless face (cyberphysical systems) is impossible without modern paradigms and technologies. Therefore, in the modern mining industry, the concept of the industrial Internet of Things and fog computing actively takes root. Support service machines are equipped with various sensor controls for constant automated monitoring which allows defining productivity during a certain time, unit operating time, identifying a technological unit. Thus, blockchain technology is key in the development of methods of authentication, data protection, and establishing a uniform entrusted information field of an enterprise.

Therefore, an urgent problem is the implementation of blockchain technology on mining facilities along with other methods of data protection. 

## 3. Methodology

In the present research, we offer approaches to the solution of the problems arising at the development of common information space of a mining enterprise. An automatic distributed dispatch system with the function of friend-or-foe identification of support service vehicles based on blockchain and digital watermarking can be a practical implementation of the given approaches.

The given system would allow identifying support service machines in the conditions of a bad connection, connecting them among themselves by a radio channel, exchanging the current condition of technological machines, and transmitting the required data to the central unit through a chain of technological units.

### 3.1. Data Flow Arising During Open-Cut Mining at a Mining Enterprise

Open-cut technologies include four basic technological stages:

- preparation of rock for the extraction;
- extraction-and-loading;
- mined rock transporting;
- unloading and storing of mined rock [4].

Each of the listed stages has a certain data flow between participants of the manufacturing process which are to be processed in a uniform information system of the mining enterprise.

The preparation of rock for extraction consists in the change of their natural condition to provide effective development of a mineral deposit. There are three basic ways of doing this:

- protection from freezing;
- hydraulic slackening or softening;
- mechanical or explosive scarifying.

The mechanical or explosive scarifying of hard rock is the most widespread on opencasts. To execute the technological process of scarifying, a higher number of support service machines and the more complex management of data flow are required at all levels of the enterprise; therefore we examine the circulation of data flow on an example of mechanical scarifying. As a rule, mechanical scarifying is carried out by specialized excavating technological cyclic machines also referred to as draglines. 

Before the beginning of the preparation process of rock to the extraction, the crews receive a development program of the site where the plan of work is specified as well as the transportation type. The transportation type of mined rock in the scarifying site defines the load on the data flow of the enterprise in the higher degree. There are two possible ways of mined rock transportation from the site: Rehandling and transportation by support service vehicles.

When following the first way, the dragline crew is engaged in the transfer of mined rock without any interaction with the dispatcher.

In the case of the second model, the dispatcher assigns support service vehicles to a dragline. The dragline crew in this case directly co-operates with the drivers of support service vehicles, bypassing the dispatcher. The inquiry on the fulfillment of technological service operations by the excavating machine crews is carried out through the dispatcher.

The stage of rock preparation for the extraction is followed by extraction-and-loading. This includes rock extraction directly from the solid and the extraction of mined rock from the muck pile, its loading onto vehicles or transporting to a dumping site. The extraction and mined rock loading are carried out by one support service machine.

On opencasts as the extracting and loading equipment, the following is used: rotating electric machines (single-bucket excavators, earth loaders, earth-moving scrapers) and continuous action machine (chain bucket excavators and rotary bucket excavators).

The mined rock loading is performed into autodump trucks, side-tipping dump cars and belt conveyors.

At this stage, the dispatcher coordinates the support service vehicles and the dragline. There are two possible ways of the organization of the loading process:

- to assign the support service vehicles to specific excavating machines;
- to assign the support service vehicles in the course of the work in an adaptive way.

In the case, if the support service vehicles are assigned to the dragline, the crews co-operate directly bypassing the dispatcher. In the case of the model of adaptive assignment, the operator assigns the support service vehicles and monitors its condition.

**Figure 1:**
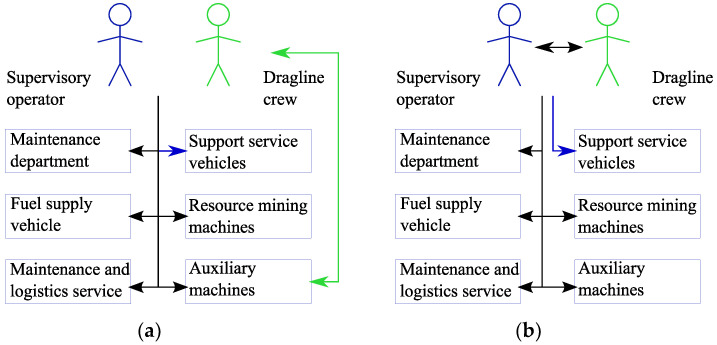
The data flow arising during the preparation of rock to the extraction and during the extraction-and-loadings: (**a**) in case of permanent assigning of support service machines to excavating machines; (**b**) in case of adaptive assigning of support service machines to excavating machines.

Figure 1 shows the data flows in both cases.

The given data flows directly influence the timeliness and efficiency of functioning of all services on the mining enterprise.

If the support service unit is out of the connection zone, the dispatcher cannot monitor its condition and send commands to realize the manufacturing process. Because of that, productivity decreases, downtime occur and material and time funds are wasted.

Besides the lack of support, the operator cannot constantly monitor the quality of the technological process, and unfair crews of support service machines frequently use this. They can exploit the technological machine in the forbidden modes for the purpose of reduction of labor input of the process of excavation. An example of it is the removal of stuck rock from the walls of a bucket by hitting it on solid objects. It can cause hard-alloy teeth breaking and the subsequent replacement would be required.

Extraction-and-loading is followed by transporting the mined rock. It is carried out in various ways depending on the mining and geological conditions of the mining technological tasks, physical and technological properties of transported rock.

Open-cut transport is designed for transporting of mined rock from mine faces to disposal points. It is a link in the technological process. The efficiency of field development depends on the accurate work of open-cut transport. The labor input of transporting is rather high, and the expenditures for actual transport and the connected tender work are 45–50%, and in some cases 65–70% of the total production cost [38,39].

At the given stage, the dispatcher monitors the current condition of support service vehicles: current spaced coordinates, fuel reserve, velocity, quantity of transported cargo, output per shift, load unloading site. On the basis of these data, the dispatcher can redistribute the places of unloading and work of accompanying services.

**Figure 2:**
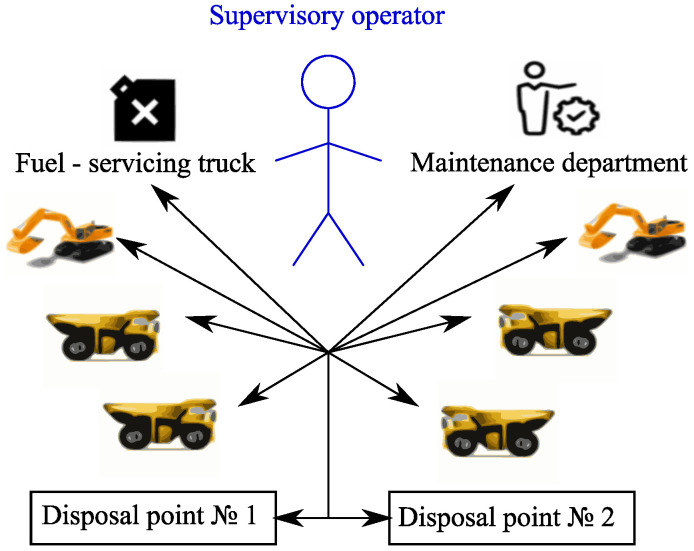
The data flow arising on the stage of mined rock transportation.

The corresponding data flow is shown in Figure 2.

**Figure 3:**
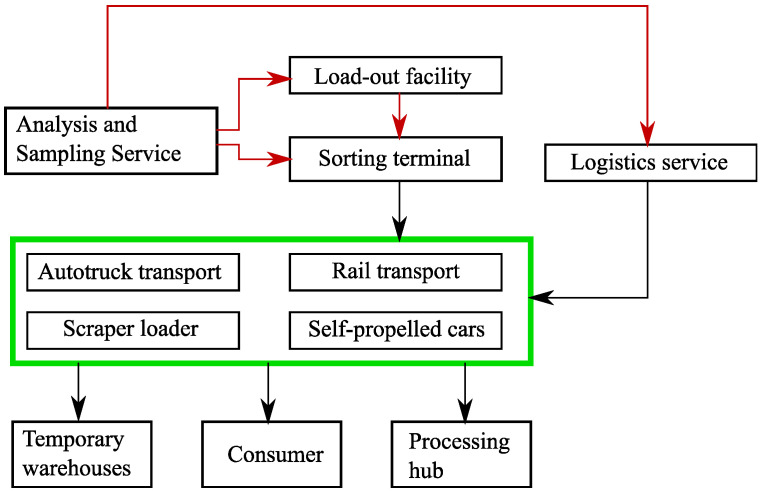
The generalized scheme of data flow at a stage of unloading and mined rock storing.

The last technological stage examined here, unloading and storing of mined rock is executed. At this stage, the dispatcher makes the decision on the unloading of support service vehicles in unloading terminals, proceeding from the current load, and whether the support service machines are available. The occurring data flow is shown in Figure 3.

**Figure 4:**
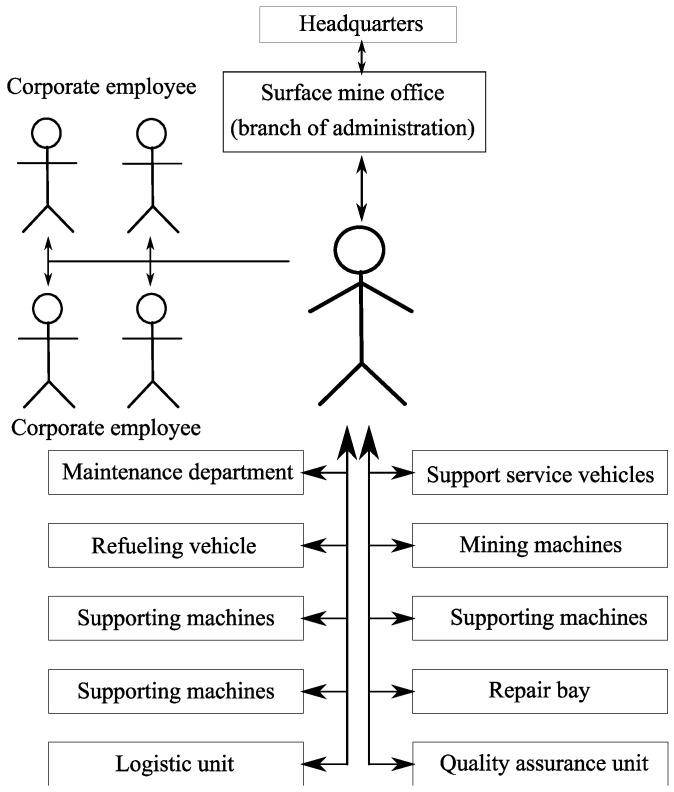
The generalized diagram of data flow.

At all stages of extraction of resources, the information from a number of sensors and sensor nodes is constantly gathered so that the enterprise dispatcher timely made decisions. A lot of data is gathered, for example, the data about fuel residual to call a fuel-servicing truck at the right time, the data about the cargo weight transported by support service vehicles. A generalized diagram of data flow is shown in Figure 4.

The presented diagram shows what data flow can be processed in common information space of a mining enterprise. We will describe possible approaches to the solution of data protection problems in the presented information field.

### 3.2. Providing of Transactions Reliability in Common Information Space of a Mining Enterprise by Means of Blockchain

The present subsection presents a new approach directed on remote quality control of exploitation of service machines in the conditions of a mining enterprise. This approach would allow us to define facts of unfair exploitation of service machines and to carry out the authentic transfer of the corresponding data in the conditions of complex terrain. For this purpose, we offer to equip each technological unit with an onboard recorder that records all transactions made by the technological unit. 

The term “transactions” is used to refer to any significant event which has occurred to technological units in the course of their work. We will select two types of such events: exceptional situations and work situations. The situations which do not occur in a regular environment of a technological unit are referred to as exclusive situations. Work situations represent the completion of any technological operation with certain parameters. If in the course of completion of the given operation, the registered parameters go beyond admissible limits, such a situation is considered exclusive.

**Figure 5:**
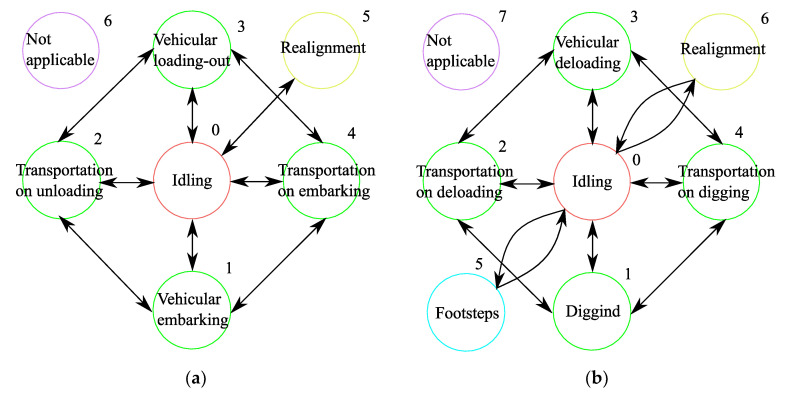
State graph: (**a**) of a transport machine; (**b**) of a dragline.

The technological process of excavation in the conditions of open-cut mining is characterized by cyclic consecutive change of working conditions of a mining machine in the following order: excavating/digging (1); transportation to unloading (2); unloading (3); transportation to excavating/digging (4). The technological processes for a dragline and service support transport are presented by means of connected graphs in Figure 5a,b accordingly. Each point of the connected graph represents a certain condition of the technological process. The interconnection between the conditions is presented by repeating transitions between the points of the state graph which characterizes the technological condition of draglines and service support transport. The state graph describes the technological process of excavation and contains the directed graph on points 1-2-3-4-1. The “Idling” condition is connected by edges to all other conditions as the forced idle time of a mining machine is possible at any moment.

Table 1 and Table 2 show the basic transactions for support service vehicles and draglines accordingly.

To store the transactions reliably, we will use a distributed ledger of the blockchain type. Various stationary objects of the infrastructure and mobile technological units will act as nodes of the corresponding network. Mobile technological units refer to various support service vehicles, maintenance machines, fuel-servicing trucks. Thus, the given blockchain is private. It covers the production of a specific enterprise or can be extended to a group of the enterprises territorially removed from each other but linked by a uniform telecommunication network.

The coordination of the ledger content will be carried out by the blockchain principle. Each node connects to the given process at the connection with any next nodes. Such an approach will allow each node of the network to have an authentic copy of the ledger even in the absence of reliable communication channels in separate sites of the network.

As an algorithm of consensus achievement, we offer to use a classical Proof-of-work algorithm. It is generally suitable irrespective of the features of a certain mine or a group of mines. An alternative variant is a newer Proof-of-authority algorithm. It ensures the essentially smaller power consumption in comparison with Proof-of-work. However, Proof-of-authority cannot be used within the limits of our problem in all cases. It is highly suitable in those mines which differ with greater connectivity of the network of the service support units. By this the following is meant. Even if the problems with connection are present on separate sites of the network, nevertheless the network does not break up into the isolated fragments. Moreover, if this network is considered as a graph, the given graph does not contain bridges. If the features of a certain mine do not guarantee the fulfillment of this feature, Proof-of-work should be used. However, the given property usually stays invariable during the long production time of the mine; therefore, a certain solution can be chosen with a view on long-term use. Naturally, for several mines connected in one network, one common decision should be chosen.

**Figure 6:**
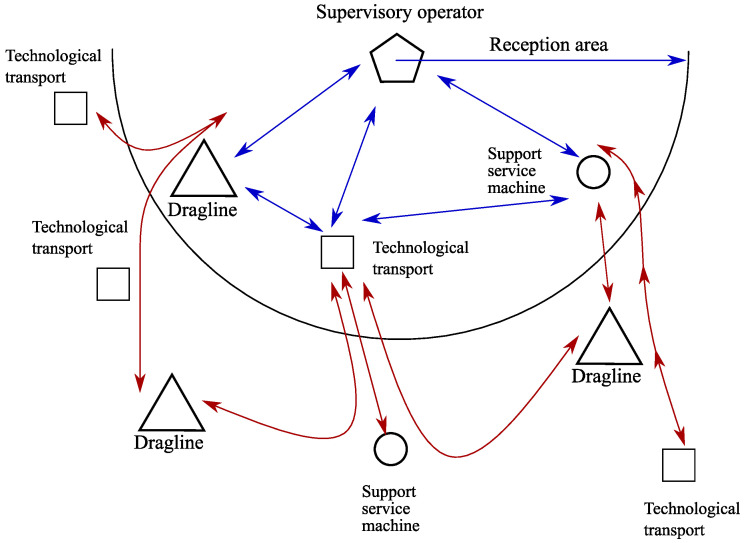
The coordination of the distributed ledger content.

Figure 6 illustrates the process of the coordination of the distributed ledger content.

The dispatching node has a special function here. It carries out the analysis of the transactions made by technological units and the decision-making on the management of the technological process. Around the dispatching node, a zone of confident reception is selected. All nodes which are in the given zone can co-operate with each other directly. The data flows of such nodes are marked by blue color. The nodes outside of the zone of confident reception transfer information through the intermediary nodes. Corresponding data flows are marked by red color. The lack of communication line between close located nodes corresponds to a situation when the connection is lost because of features of the terrain of the area.

It is necessary to note, that the given diagram contains one potential problem. It is designed for a fully connected network that does not contain isolated nodes. However, the features of the mining production process described in Section 3.1 prove that such nodes are possible. In this case, the method of the coordination of the register content should be specified.

We suggest solving the given problem by means of organizational measures. Generally, the time of the complete lack of connection of a separate technological unit does not exceed several minutes. Therefore, it is enough to reduce the time ranges between the movement of mobile units when it is possible. First of all, it is a question of repair transport. And those service support units which could usually lose connection for some time are now always accessible by any other service support unit. The requirement to maximum time during which the service support unit can remain without connection is a limitation of our approach. However, this requirement is met at all mines known to us; therefore, the given simple solution is effective.

Now, let us describe the format of transactions in more detail. They include identification data and the data produced by sensors of technological units. These data depend on the type of a technological unit.

The most numerous type of technological units on a mining enterprise is truck transport. It ensures the delivery of mined rock from the extraction site to the loading site. A large number of transport machines allow one to ensure uninterrupted operation of the given process. The monitors and sensors of the truck transport allow one to read the following basic types of data: current co-ordinates, current velocity, the mass of transported cargo, fuel residual, the pressure in tires, angles of inclination and acceleration, the control of the gear shifter.

The scarifying of hard rock is carried out by means of draglines. For the given type of technological units, first of all, it is necessary to monitor the following data: current co-ordinates, bearing angle, angles of orientation and acceleration, angular velocity of the platform, current technological condition, the type and quantity of the executed duty cycles, time characteristics of cycles. Mainly, on the basis of the values of linear acceleration, angular velocity and spatial co-ordinates, the exploitation quality of an excavator is monitored, and the duty cycles, productivity, the current condition, and time characteristics are calculated.

**Figure 7:**
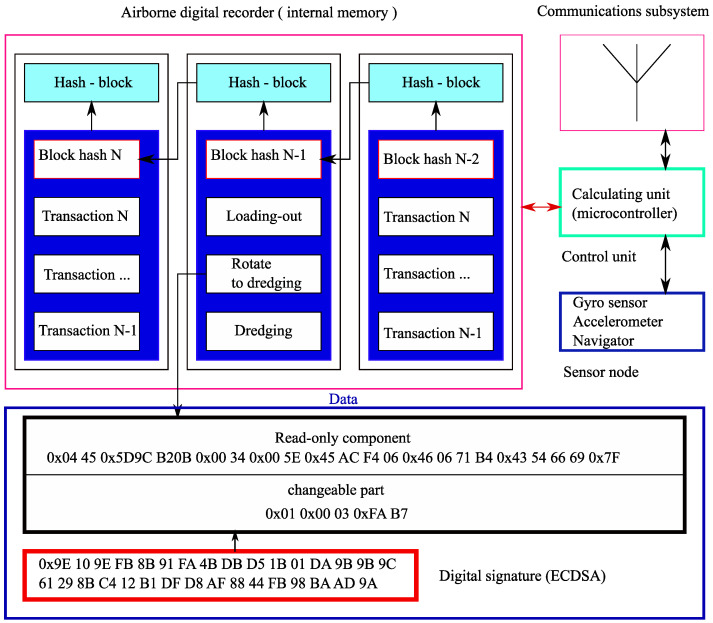
Formation of a block of transactions.

Figure 7 shows an example of the formation of a block of transactions at the level of a separate technological unit.

### 3.3. Providing of Authentication of Sensor Data Produced by Support Service Vehicles, by Means of Digital Watermarking

As an additional level of protection, in the present research, it is offered to use digital watermarks. The purpose is the binding of the data produced by technological units to the source of its origin.

Earlier, we presented the data structure describing the format of transactions registered in the system. The elements of the data entering into this structure could be divided into two types: non-modifiable and modifiable. The elements of the data of the first type cannot be changed, they are produced automatically and irrespective of external factors. They include the record identifier, the machine identifier, etc. The data elements of the second type represent indications of various sensors. They could be altered; however, these alterations should be reversible.

**Figure 8:**
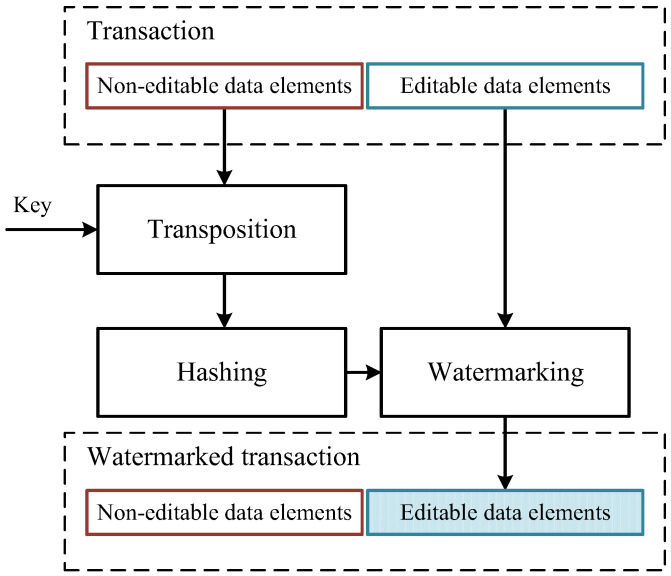
The model of digital watermark embedding into the data produced by technological units.

We propose to use the modifiable data elements for the embedding of reversible digital watermarks. The corresponding model is presented in Figure 8.

In the given model, a digital watermark is formed on the basis of non-modifiable data elements and a confidential key. They are combined using a simple mixing transformation, then the result of the combination is hashed. The received hash-code is also a digital watermark. It is embedded in modifiable data elements using a reversible function of embedding.

The verification of digital watermarks is carried out on the side of the dispatcher after the technological units completed the work. It is advisable to use this approach when a small number of units of equipment participate in the technological process. In this case, digital watermarks would be an additional way of protection of the generated distributed register from attempts to compromise it.

## 4. Computing Experiment and the Discussion of the Results

### 4.1. Purpose of the Experiment

To implement the offered solutions in a mining enterprise, it is required to equip support service units with additional hardware modules which implement functions of preprocessing and protection of transmitted data. As a matter of fact, it is a question of IoT-system development. In this connection, it is necessary to pay considerable attention to questions of energy-efficiency. For the Internet of Things systems, the decrease of power consumption is one of the key problems that many researchers are engaged with. For example, in [40] the effect of enciphering algorithms on synchronization and energy consumption in IoT applications is investigated. Similar questions are discussed in [41] as well.

Among the offered solutions, the most resource-consuming is blockchain technology which is based on repeated execution of fulfillment of hashing operation. The digital watermark embedding is simpler in terms of computing and does not require a considerable quantity of operations. Therefore, the given section is devoted to the computing experiments connected to blockchain technology.

The purpose of the given experiments consists in the determination of the possibility of technical realization of the approach described in Section 3.2 and based on blockchain. For this purpose, we study the hardware capabilities of modern microprocessor control units (MCU) on the basis of which modern intelligent electronic devices are built, such as sensor nodes, data acquisition systems and data processing systems, and control systems.

The software architecture of intelligent devices has the following feature. The control microprogram functions in a multithreading or pseudo-multithreading mode organized by operating systems or specialized libraries (continuations, coroutines, finite state machines, Duff’s devices). Such an architecture solution is connected to the necessity of constant multithreading task execution and processing of great volumes of data.

MCU should possess computing capacity sufficient for the work of an operating system and software. In the studied case, the key task is to calculate the hash-values according to the SHA-256 algorithm. The acceptable time of fulfillment of the given task should not exceed 1 ms. The specified time is explained by the feature of multithreading (pseudo-multithreading) program operation.

The task planner in the majority of operating systems for MCU functions with the discreteness of 1 ms.

If the time of hash-value calculation exceeds 1 ms, the operating system task planner would have to interrupt (displace) the current calculation operation, carry out the next task and after its completion to return to the initial task. The features of the hashing algorithm realization would cause the increased load of operating memory in MCU, and in the worst case, the data can be garbled.

### 4.2. Organization of the Experiment

The experiment on research of hardware speed is organized as follows.

First, two common MCU architecture are chosen:

- ARM Cortex;
- Atmel AVR 8.

The given architectures are widely popular with the developers of radio equipment. MCU of the Atmel AVR architecture are used in simple radio-electronic devices. They have an 8-bit core and are used to carry out simple tasks that do not require numerous calculations. Also, they are often used as a co-processor.

ARM Cortex architecture is in great demand with developers thanks to a wide range of accessible families and the lineup differing with functional, performance and price. ST microelectronics Company produces 12 basic families of microcontrollers with ARM architecture (STM32L0/L1/L4/L5/F0/F1/F2/F3/F2/F4/F7/H7).

Secondly, eight models of MCU were chosen that have the specified architecture.

Cortex-M architecture (manufactured by ST microelectronics):

- STM32F722ZE,
- STM32F407VET6,
- STM32F303CCT6,
- STM32F103C8T6,
- STM32F100RBT6B,
- STM32F051R8T6,
- STM32F030F4P6.

AVR 8 architecture (manufactured by Microchip):

- Xmega32A4U;
- AtMega328.

The nomenclature of the tested MCU is determined by the features which are inherent in each series. The microcontrollers of series L0, L1, L4 and L5 have small energy-consumption and are focused on application in the systems with high requirements to energy-saving, therefore, were not examined. MCU of series F0, F1, F3, F4 and F7 have sufficient performance and functionality, good operating characteristics as well as good availability. These MCU families are optimum for application in data-processing systems and the development of control systems. Series F2 and H7 are superfluous for the examined problem.

Further, for the immediate experiment, the integrated development environment and libraries of cryptographic functions corresponding to them were chosen:

- IAR Embedded Workbench for ARM, libraries STM32 STM32CryptographicV3.1.1 / STM32 CryptographicV3.0.0;
- AVR Studio, AVR-Crypto-Lib library.

At last, the volume of hash information is accepted as equal to 1500 bytes, considering the minimum length of a Jumbo frame for the Ethernet network (standard IEEE 802.3).

The procedure of the experiment included the following steps.

**Step 1.**  Project adjustment, installation of necessary libraries, insertion of the unified tested code. Installation of various strategies (levels) of the program optimization (optimization on velocity, optimization on the size of the controlling microprogram).
**Step 2.**  Adjustment of the clock system for the maximum operating frequency for each model of the microcontroller.
**Step 3.**  Adjustment of hardware blocks for calculation of execution time of the SHA-256 hash algorithm:
  - for STM32 microcontroller, a DWT counter (Data Watchpoint and Trace unit) was used, as well as a TIM2timer, and input-output ports;
  - AVR microcontrollers were tested in a simulator (the integrated development environment AVR Studio rather than IAR Embedded Workbench allows to precisely evaluate the execution time of the microprogram).

**Step 4.**  Program start through the programmator:
  - STlink-v3 debugger for STM32microcontroller;
  - a simulator for an AVR microcontroller.

**Step 5.**  Data collection of execution time of the program:
  - for microcontrollers STM32F407VET6, STM32F303CCT6, STM32F103C8T6, STM32F100RBT6B directly from the DWT counter;
  - for STM32F051R8T6 microcontroller directly from TIM2 timer-counter;
  - for STM32F030F4P6 microcontroller indirectly by means of DS Logic logic analyzer (through gauging of the installation time and logic state reset of input-output ports);
  - for single-crystal AVR microcomputer by means of the simulator.

The received results are shown in Table 3, Table 4 and Table 5.

The analysis of the results of the carried out experiment allows us to draw the following conclusions.

1. The efficiency of the libraries CryptographicV3.0.0 and CryptographicV3.1.1 differs in productivity on average by a factor of 1.5, except MCU STM32F407. CryptographicV3.0.0 library has the best results for MCU STM32F407 with optimization on velocity.
2. CryptographicV3.1.1 library has considerably better optimization on velocity and the size of the program than CryptographicV3.0.0 with the MCU of younger families (F0, F1, F3).
3. The productivity gain at different strategies of program optimization starts to be developed with MCU STM32F103. For this MCU, the optimization on velocity and the size of the microprogram allows us to raise the performance by a factor of 1.3. With STM32F303MCU, the optimization strategy on velocity gives a considerable gain of performance (by a factor of 1.83), thus the size of the program on average increases only by 7–10%.
4. The conditions of optimum speed meet the requirements of MCU starting with the families M32F3, STM32F4 and higher.
5. MCU with AVR 8 architecture does not meet the set requirements of the performance.

Along with the hash function in the blockchain, the digital signature algorithms are used. They are essentially less often addressed; however, they are enough resource-consuming themselves. Therefore, it was expedient to evaluate the operation speed of such algorithms as well. For experiments we chose the RSA and ECDSA algorithms, presented in STM32 CryptoLib library of v3.0.0 version and STM32 CryptoLib library of v3.11 version. The experiment was carried out on three MCU models which showed the greatest efficiency in the previous experiment: STM32F722, STM32F407, STM32F303.

The results received for the RSA algorithm, are brought in Table 6 and Table 7.

As a result of the experiment, the following is determined:

1. RSA algorithm is labor-consuming enough and requires a considerable amount of processor time.
2. The feature of the RSA algorithm realization in the CryptoLib library does not allow dividing the signing process into several stages.

Thus, RSA has proved to be a slow algorithm that insufficiently effectively functions on middle-level MCU STM32.

The next experiment was devoted to the ECDSA algorithm. The results received for the given algorithm, are brought in Table 8 and Table 9.

As a result of the experiment, the following is determined:

1. The run time of the given algorithm is lower than the RSA has; however, the productivity gain is not proportional, the productivity gain fluctuates in the range from 5 to 12%.
2. The feature of realization of the ECDSA algorithm in the CryptoLib library allows dividing the signing process into several stages.

Therefore, out of the two examined options, the ECDSA algorithm is preferable.

Thus, MCU of series STM32F303 and STM32F4 possess the necessary performance to build on their basis the hardware modules realizing the blockchain-based approach offered in Section 3.2. The given approach allows us to ensure the reliability of transactions in the common information space of a mining enterprise in the conditions of possible loss of connection between the control center and separate service support units.

Practical realization of the given approach and its implementation as a part of common information space of a mining enterprise will be made during further studies.

## 5. Conclusions

The given paper is devoted to the problem of providing the reliability of data on the production course on a mining enterprise in the conditions of possible loss of connection between the control center and separate service support units. This is a part of a larger-scale task of the development of the common information space of a mining enterprise. To solve the given task, we offered several new approaches to the protection of the data generated by service support machines. Our first approach ensures the reliability of transactions in the common information space of a mining enterprise by means of blockchain technology. We used private blockchain with the consensus algorithm of the Proof-of-work type. Besides, we have presented the case when it is expedient to use the Proof-of-authority type of algorithm differing by higher energy-efficiency. To describe the actions made by support service machines, we used the concept of a transaction. We defined the format of a transaction and formed a list of transactions for two basic types of service support machines. A limitation of the given approach is the short duration of connection loss between service support units. Our second approach ensures the authentication of the sensor data developed by service support machines by means of digital watermarks. This approach supplements the blockchain and ensures additional data binding to the conditions of their generating. We introduced the division of data elements as a part of each transaction on edited and not edited ones. We have suggested forming a digital watermark on the basis of not edited data elements and a secret key and embedding it into the edited data elements. At last, we conducted a computing experiment with common models of microcontrollers. During the given experiment, we implemented the basic cryptographic algorithms necessary for the building of the blockchain and evaluated the efficiency of their work when using various strategies of optimization. The results of the experiment confirmed the realizability of our approaches.

The further work will consist in the development of the offered approaches and their implementation in mining enterprises. Besides, the results of the conducted research can be used for data protection in other areas. For example, a promising direction is the protection of transactions in vehicular ad-hoc networks.

## Figures and Tables

**Table 1:** Transactions for support service vehicles.

Exceptional Situations	Working Situations
Inner tube deflation	Embarking
**Wheel status**: failure; **status code**: 0x0012; **state argument**: 0xFFFE (in one wheel pressure is below normal).	**Loading status**: successfully; **weight cargo**: 0x034C88 (weight 216.2 tons); **loader ID**: 0x346; **unloading area identifier**: 0x01B; **generalized status**: 0x00 (no violations).
Excessive angular orientation	Loading-out
**Orientation status**: failure; **status code**: 0x0012 (roll angle excess); **state argument**: 0xC20CCCCD	**Loading status**: failure; **cargo weight**: 385 400 kg; **unloading area identifier**: 0x0F15 (unloading in the wrong place); **status argument**: 0x067 (premature discharge); **generalized status**: 0x00 (no violations).
Hardware fault	Access to destination
**Equipment status**: successful; **status code**: 0x0000; **state argument**: 0x0000 (equipment in good condition).	**Item achievement status**: successful; **destination ID**: 0x0145; **curvature of trajectory**: 0xF1; medium speed: 0x14; **steering rack angle**: 0x0009; **generalized status**: 0x00 (everything is fine).
Low fuel level	Deallocation
**Fuel status**: failure; **status code**: 0x0001; **state argument**: 0x73 (fuel remaining 115 L).	**Temporary destination ID**: 0x0018; **status of achievement of point**: successful; **status of the transported freight**: 0x0034; **target identifier**: 0x18A; **curvature of trajectory**: 0x05; **middle speed**: 0x12; **steering rack angle**: 0x01; **generalized status**: 0x05 (successful but with allowed overload).
Approach/stay in the restricted area	Staying
**Location status**: successful; **status code**: 0x0001; **state argument**: 0x00 (out of zone).	**Generalized status**: 0x0001; **status code**: 0x01 (premature unloading); **state argument**: 0x407 (stalled).
Locating people and vehicles in the restricted area	
**Location status**: failure; **status code**: 0x0015; **state argument**: 0x0107 (7 people in dangerous proximity).	
Transmission Status (temperature, oil level)	
**Transmission status**: failure; status code: 0x0026; **state argument**: 0x4251999A (slight oil heating).	
Other contingency	
**Situation status**: failure; **status code**: 0x0036; **state argument**: 0x3138 (overload 12.6 tons); **state argument extension**: 0x315 (loader ID).	

**Table 2:** Transactions for draglines.

Exceptional Situations	Working Situations
Excess of angular speed	Platform rotation
**Orientation status**: successful; **status code**: 0x0000; **state argument**: 0x00.	**Turn status**: successful; **status code**: 0x0003 (dredging); **state argument**: 0xFAB7 (dredging, average velocity-12.6 deg).
Over acceleration	Digging
**Acceleration status**: successful; **status code**: 0x0000; **state argument**: 0x00.	**Digging status**: successful; **status code**: 0x0002; **state argument**: 0x12A7.
Exceeding allowed orientation angles	Deloading
**Orientation status**: failure; **status code**: 0x0075 (pitching displacement); **state argument**: 0x0330 (current orientation 3.16 deg).	**Deloading status**: successful; **status code**: 0x0004; **state argument**: 0x87FA.
Hardware fault	Transportation
**Hardware status**: failure; **status code**: 0x0175 (auxiliary transformer failure); **state argument**: 0x0042.	**Transportation status**: successful; **status code**: 0x0007; **state argument**: 0x0000 (no violations).
Approach / stay in the restricted area	Secondary operations
**Entry status**: failure; **status code**: 0x00FC entry to restricted area); **state argument**: 0x0012 (gib arm).	**Operation status**: successful; **status code**: 0x0001 (cleanup); **state argument**: 0x0000.
Finding people and vehicles in the restricted area	Current status
**Location status**: successful; **status code**: 0x0000; **state argument**: 0x00 (objects outside the zone).	**Current status**: failure; **status code**: 0x0014 (fault time); **state argument**: 0x0012 (waiting for auxiliary vehicles).
Power supply failure	
**Power supply system**: successful; **status code**: 0x0000; **state argument**: 0x0000 (normal work).	
Generalized state:	
**Current status**: successful, **status code**: 0x0000, **state argument**: 0x0000 (normal work).	

**Table 3:** Results of testing of Cortex-M MCU architecture with STM32 CryptographicV3.0.0 library (SHA-256 algorithm).

MCU	Core	Fmcu	Strategy of Optimization	Cycles/Time, ms	Size of the Program	Difference	
						Time	Size
STM32F722	M7	216	Speed	68 463/0.316	13 996	3.23	~1
			Size	211 255/1.022	14 205		
STM32F407	M4	168	Speed	77 770/0.463	14 562	3.68	~1
			Size	239 250/1.24	14 496		
STM32F303	M4	72	Speed	100 063/1.38	15 801	3.07	~1
			Size	369 115/5.12	15 785		
STM32F103	M3	72	Speed	97 457/1.353	14 353	3.76	~1
			Size	364 112/5.075	14 271		
STM32F100	M3	24	Speed	74 794/3.116	14 271	3.15	~1
			Size	236 112/9.838	14 226		
STM32F051	M0	48	Speed	184 216/3.837	15 955	1.93	1.04
			Size	356 888/7.435	15 292		
STM32F030	M0	48	Speed	184 256/3.838	14 875	1.93	1.04
			Size	356 904/7.435	14 179		

**Table 4:** Results of the test of MCU with Cortex-M architecture with the STM32 CryptographicV3.1.1 library (SHA-256 algorithm).

MCU	Core	Fmcu	Strategy of Optimization	Cycles/Time, ms	Size of the Program	Difference	
						Time	Size
STM32F722	M7	216	Speed	89 115/0.412	15 736	1.55	1.12
			Size	123 407/0.571	13 937		
STM32F407	M4	168	Speed	100 754/0.599	14 795	1.153	~1
			Size	116 565/0.693	13 768		
STM32F303	M4	72	Speed	119 349/0.887	16 108	1.86	1.07
			Size	149 158/1.652	14 991		
STM32F103	M3	72	Speed	116 742/1.62	14 676	1.30	1.08
			Size	152 334/2.115	13 490		
STM32F100	M3	24	Speed	97 816/4.07	14 607	1.16	1.08
			Size	113 609/4.73	13 445		
STM32F051	M0	48	Speed	194 676/4.05	15 861	1.05	1.08
			Size	203 392/4.237	14 568		
STM32F030	M0	48	Speed	194 695/4.056	14 781	1.04	1.09
			Size	203 389/4.237	13 455		

**Table 5:** Results of the test of MCU with AVR 8 architecture (SHA-256 algorithm).

MCU	Core	Fmcu	Strategy of Optimization	Cycles/Time, ms	Size of the Program	Difference	
						Time	Size
XMEGA32A4U	AVR	32	Speed	185 547/5.802	7 566	1.08	2.14
			Size	200 574/5.813	3 480		
Atmega328	AVR	20	Speed	181 232/9.061	7 180	~1	2.32
			Size	182 641/9.132	3 094		

**Table 6:** Results of testing of Cortex-M MCU architecture with STM32 CryptographicV3.0.0 library (RSA algorithm).

MCU	Core	Fmcu	Strategy of Optimization	Cycles/Time, ms	Size of the Program	Difference	
						Time	Size
STM32F722	M7	216	Speed	14 560 925/67.4	105 127	1.24	1.52
			Size	18 108 317/83.8	69 138		
STM32F407	M4	168	Speed	13 871 886/82.1	96 202	1.45	1.49
			Size	20 208 885/120.2	64 432		
STM32F303	M4	72	Speed	15 492 341/215.1	82 174	~1	1.26
			Size	23 780 397/330.0	65 725		

**Table 7:** Results of the test of MCU with Cortex-M architecture with the STM32 CryptographicV3.1.1 library (RSA algorithm).

MCU	Core	Fmcu	Strategy of Optimization	Cycles/Time, ms	Size of the Program	Difference	
						Time	Size
STM32F722	M7	216	Speed	11 126 863/51.5	84 515	2.33	1.44
			Size	25 995 872/120.3	58 519		
STM32F407	M4	168	Speed	15 126 863/89.9	81 920	1.60	1.34
			Size	24 382 272/145.6	60 779		
STM32F303	M4	72	Speed	16 970 675/235.7	82 714	1.86	1.07
			Size	28 382 439/394.4	62 059		

**Table 8:** Results of testing of Cortex-M MCU architecture with STM32 CryptographicV3.0.0 library (ECDSA algorithm).

MCU	Core	Fmcu	Strategy of Optimization	Cycles/Time, ms	Size of the Program	Difference	
						Time	Size
STM32F722	M7	216	Speed	11 932 102/55.2	105 027	1.13	1.44
			Size	14 037 648/65.0	72 936		
STM32F407	M4	168	Speed	12 137 058/72.2	96 202	1.13	1.49
			Size	13 718 011/81.6	64 432		
STM32F303	M4	72	Speed	13 358 941/185.2	82 174	1.20	1.52
			Size	16 064 863/232.0	65 725		

**Table 9:** Results of the test of MCU with Cortex-M architecture with the STM32 CryptographicV3.1.1 library (ECDSA algorithm).

MCU	Core	Fmcu	Strategy of Optimization	Cycles/Time, ms	Size of the Program	Difference	
						Time	Size
STM32F722	M7	216	Speed	12 468 117/57.7	105 027	1.13	1.44
			Size	15 437 202/71.4	72 936		
STM32F407	M4	168	Speed	12 896 025/76.7	81 920	1.13	1.47
			Size	14 631 794/82.8	60 779		
STM32F303	M4	72	Speed	14 206 394/197.7	82 714	1.20	1.53
			Size	17 124 143/237.3	62 059		
